# Predictive value of the atherogenic index of plasma for chronic total occlusion before coronary angiography

**DOI:** 10.1002/clc.23565

**Published:** 2021-03-09

**Authors:** Tong Liu, Jinghua Liu, Zheng Wu, Yun Lv, Wenzheng Li

**Affiliations:** ^1^ Department of Cardiology, Beijing Anzhen Hospital Capital Medical University, Beijing Institute of Heart, Lung and Blood Vessel Diseases Beijing Chaoyang China

**Keywords:** atherogenic index of plasma, chronic total occlusion, coronary angiography, diagnosis, Gensini score

## Abstract

**Background:**

The atherogenic index of plasma (AIP) is calculated by logarithmic transformation of the ratio of triglyceride (TG) and high‐density lipoprotein cholesterol (HDL‐C) concentrations. Although previous studies have demonstrated that the AIP is associated with coronary artery disease, its association with chronic total occlusion (CTO) requires elucidation.

**Hypothesis:**

We hypothesized that the AIP would have diagnostic value in cases of CTO and could be used to predict adverse events.

**Methods:**

This study involved 1131 inpatients who underwent coronary angiography. Data on demographic and clinical characteristics, coronary artery stenosis rated by the Gensini score, and clinical assessment by the Global Registry of Acute Coronary Events and thrombolysis in myocardial infarction (TIMI) scores were collected by cardiovascular doctors. Serum AIP values were evaluated by logarithmic transformation of the ratio of TG and HDL‐C concentrations. The correlations of AIP values with clinical parameters were assessed, and receiver‐operating characteristic curves were constructed for CTO diagnosis.

**Results:**

Overall, 1131 inpatients were assigned to the CTO (*n* = 398) and control (*n* = 733) groups. Compared with the control group, the CTO group showed a significantly higher AIP (p < .05). The AIP was positively correlated with body mass index, the TIMI score, the Gensini score, and stent length and was effective for the diagnosis and risk assessment of patients with CTO. Multivariate logistic regression analyses revealed that the AIP was an independent risk factor for CTO. The findings suggest that the AIP could predict the presence of CTO and disease severity.

## 1 INTRODUCTION

Chronic total occlusion (CTO) is defined as the complete obstruction of thrombolysis in myocardial infarction (TIMI) Grade 0 flow for more than 3 months, which is frequently encountered during coronary angiography in patients with coronary artery disease (CAD).^1^ In comparison with incomplete stenosis, CTO is associated with a significantly higher rate of major adverse cardiovascular events. The advancements in materials and technical methods have incrementally improved the procedural success rate for CAD. However, the revascularisation rate of CTO remains low, and revascularisation is unsuccessful in approximately 20%–35% of CTO cases, even when the procedures are performed by experienced operators.^2^ With its high prevalence and low revascularisation rate, CTO remains a major challenge in current interventional cardiology.

Dyslipidaemia is one of the classic factors of CAD that contribute to the progression of atherosclerosis. Preventive management protocols can prominently alter cardiovascular outcomes.^3^ As therapeutic targets, an elevated triglyceride (TG) concentration and a low high‐density lipoprotein cholesterol (HDL‐C) concentration show a strong predictive value for cardiovascular events, reflecting the balance between the atherogenic and anti‐atherogenic states. The atherogenic index of plasma (AIP), which is determined by logarithmic transformation of the ratio of TG and HDL‐C concentrations, was first described in 2001 as a sensitive marker of CAD.^4^ Several groups have reported that the AIP is associated with the onset of CAD and all‐cause death. Cai et al. revealed that the AIP is a strong and independent predictor for CAD.^5^

Despite the body of evidence indicating the association between the AIP and CAD, the value of the AIP as a predictive biomarker for the severity of coronary syndrome remains controversial, particularly in CTO, and the association between the AIP and CTO has not been established well. Therefore, we investigated the association of the AIP with CTO development and deduced the usefulness of the AIP for the diagnosis and risk assessment of CTO in clinical practice.

## 2 METHODS

### 2.1 Study cohort

Overall, 1131 inpatients with chest pain and suspected acute coronary syndrome who had undergone coronary angiography from January 2018 to December 2018 at The Anzhen Hospital were consecutively enrolled in this study. The patients were assigned to the CTO (*n* = 398) or non‐CTO (control, *n* = 733) group. CTO was defined as follows: (1) an occlusion lasting for more than 3 months based on the first onset of angina pectoris, previous angiogram findings, and previous infarction, and (2) TIMI Grade 0. Non‐CTO was defined as stenosis of at least 50% of the luminal diameter in at least one major coronary artery branch.

### 2.2 Procedure

Data regarding the patient's sex, age, medical history, smoking status, body mass index (BMI), blood pressure, heart rate, complete blood count, serum cholesterol and homocysteine concentrations, and medication history were collected for all participants. The diagnostic criteria for the classic risk factors, including dyslipidaemia,^6^ hypertension,^7^ and type 2 diabetes mellitus,^8^ were based on authoritative international guidelines. The Global Registry of Acute Coronary Events (GRACE) and TIMI scores were calculated from the clinical data.^9^ Fasting venous blood was drawn from all participants and analyzed by an automated biochemical analyzer. The AIP was calculated by logarithmic transformation of the ratio of TG and HDL‐C concentrations (log_10_ [TG/HDL‐C]).

### 2.3 Coronary angiography

All participants underwent coronary angiography, which was conducted by an experienced team of cardiologists. The cardiologists were blinded to the groups when collecting the data. The severity of coronary artery stenosis was quantified by the Gensini score: 0, no stenosis; 1, <25% stenosis; 2, 25%–50% stenosis; 4, 50%–75% stenosis; 8, 75%–90% stenosis; 16, 90%–99% stenosis; and 32, 100% stenosis. The scores were multiplied by a factor based on the position of the lesion, for example, 5 for the left main coronary artery, 2.5 for the proximal left circumflex coronary artery (LCX) or proximal left anterior descending coronary artery (LAD), 1.5 for the mid‐region of the LAD, 1.0 for the distal right coronary artery (RCA) or posterolateral branch of the LAD, first diagonal branch, and mid‐distal region of the LCX or obtuse branch, and 0.5 for the other segments.^10^ The segments of the coronary arteries were defined according to the American Heart Association guidelines. Specifically, the proximal RCA was defined from the ostium to the first large right ventricular branch or the first bend. The mid‐RCA was defined from the end of the proximal RCA to the second bend. The distal RCA was defined from the end of the mid‐RCA to the origin of the posterior descending branch. The proximal LAD was characterized as extending from the end of the left main coronary artery to the first septal or first diagonal branch. The mid‐LAD was characterized as extending from the end of the proximal LAD to the point where the LAD forms an angle. The distal LAD was used to refer to the remaining segments of the LAD. The proximal LCX was defined from the ostium to the origin of the obtuse marginal branch. The mid‐proximal LCX was defined as extending from the end of the proximal LCX to the end of the LCX.^11^

### 2.4 Statistical analyses

Continuous variables are reported as the means ± SD for normally distributed data or medians and quartiles (Quartile 1; Quartile 3) for non‐normally distributed data. Discrete variables are expressed as frequencies and percentages and were compared using the χ^2^ test. The correlations between inflammatory cytokines and coronary stenosis as well as clinical parameters were assessed using the Spearman rank‐order test. Receiver‐operating characteristic (ROC) curves were constructed, and the areas under the curves (AUCs) were calculated to obtain the cutoff values for CTO diagnosis.

Univariate and multivariate logistic regression analyses were performed to assess the strength of the effect of the AIP on the risk of CTO, and data are expressed as odds ratios (ORs) with 95% confidence intervals (95% CIs). A two‐sided p‐value <.05 was considered statistically significant. The statistical computations were performed using the SPSS software, version 19.0 (IBM Corp., Armonk, NY, USA).

## 3 RESULTS

### 3.1 Baseline demographic and clinical characteristics

In the CTO group, angiographic coronary stenosis with one‐vessel involvement was seen in 84 patients (21.1%); two‐vessel involvement, in 119 patients (21.9%); and three‐vessel involvement, in 195 patients (49.0%). The incidence of hyperlipidaemia, type 2 diabetes mellitus, and smoking was significantly higher in the CTO group than in the control group (p < .05). Similarly, the CTO group showed significantly higher BMI, white blood cell (WBC) counts, and brain natriuretic peptide and C‐reactive protein concentrations than the control group (all p *<* .05). However, no significant intergroup differences in hypertension, stroke, and history of percutaneous coronary intervention were detected between the groups. In addition, the patients in the CTO group were older and predominantly male than those in the control group (all p > .05; Table 1).

**TABLE 1:** Baseline characteristics of demographic and clinical findings

Factor	CTO group	No‐CTO group	χ^2^	p
	(*n* = 398)	(*n* = 733)		
Male[n(%)]	348 (87.4)	626 (85.4)	0.893	.345
Age(*years*)	65.2 ± 10.6	63.1 ± 11.3	−3.075^a^	.08
BMI(Kg/m^2^)	25.52 ± 3.06	24.50 ± 3.27	2.219^a^	.028
Smoking[n(%)]	227 (57.0)	350 (47.7)	8.901	.003
Hyperlipidaemia[n(%)]	246 (61.8)	361 (49.2)	16.363	<.001
Hypertension[n(%)]	189 (47.5)	320 (43.5)	1.53	.216
Type 2 diabetes mellitus [n(%)]	193 (48.5)	266 (36.3)	15.93	<.001
History of coronary artery disease[n(%)]	55 (13.8)	73 (10.0)	3.829	.05
strok[n(%)]	31 (7.8)	58 (7.9)	0.005	.941
History of PCI[n(%)]	77 (19.3)	141 (19.2)	0.002	.964
SBP(mmHg)	133.8 ± 19.2	126.7 ± 21.6	5.679^a^	<.001
DBP(mmHg)	77.7 ± 11.5	76.4 ± 12.2	1.779^a^	.075
HR (bpm)	73.1 ± 32.6	70.7 ± 10.9	−1.839^a^	.066
LVEDD (mm)	50.8 ± 6.7	50.0 ± 6.5	−1.626^a^	.104
EF(%)	58.0 (47.3，64.0)	61.0 (54.0，68.0)	−4.941	<.001
WBC(10^12^/L)	7.38 (6.10,9.34)	6.85 (5.79，8.25)	−4.437	<.001
RBC(10^12^/L)	4.37 ± 0.68	4.30 ± 0.62	−1.719^a^	.086
HGB(g/L)	136.0 (121.0,150.0)	134 (121，146)	−1.256	.209
PLT(10^9^/L)	203.3 ± 55.6	202.1 ± 59.7	−0.337^a^	.736
TP(g/L)	66.6 ± 6.8	67.5 ± 6.5	2.051^a^	.04
Glu(mmol/L)	6.19 (5.31,8.27)	5.89 (5.02，7.89)	−1.507	.132
TC(mmol/L)	4.16 ± 0.97	3.92 ± 0.87	−2.587^a^	.01
TG(mmol/L)	1.9 (1.4，2.5)	1.27 (1.01，1.59)	−14.121	<.001
HDL(mmol/L)	0.92 (0.79，1.12)	1.27 (1.01，1.59)	−6.895	<.001
LDL(mmol/L)	2.51 ± 0.84	2.63 ± 0.88	2.268^a^	.023
LPa(mmol/L)	0.05 (0.02,0.25)	0.08 (0.02，0.22)	0.894	.401
Hcy(mmol/L)	19.5 (15.8，26.0)	21.05 (16.48，28.05)	−1.735	.083
UA(mmol/L)	436.0 ± 116.7	422.8 ± 105.5	−1.926^a^	.054
BNP(pg/ml)	375.5 (167.2，1163.7)	196.4 (73.9，536)	−3.676	<.001
CRP(mg/L)	4.1 (1.5，11.8)	2.94 (1.06，8.94)	−3.419	<.001
GRACE score	140 (118，164)	129 (112，153)	−4.174	.032
TIMI score	4 (3，4)	3 (3，4)	−2.322	.041
Gensini score	85 (63，114.1)	51 (39，70.5)	−15.445	<.001
AIP	0.361 (0.332，0.393)	0.322 (0.293，0.341)	−14.632	<.001
Angiography				
1‐vessel	84 (21.1)	161 (22.0)	0.112	.738
2‐vessels	119 (29.9)	240 (32.7)	0.962	.327
3‐vessels	195 (49.0)	332 (45.3)	1.42	.233
CTO‐vessel				
LM	6 (1.5)	—	—	—
LAD	143 (35.9)	—	—	—
LCX	53 (13.3)	—	—	—
RCA	196 (49.2)	—	—	—
Stent				
0	16 (4.0)	45 (6.1)	2.27	.132
1	123 (30.9)	296 (40.4)	9.934	.002
2	121 (30.4)	262 (35.7)	3.286	.07
3	79 (19.8)	85 (11.6)	14.171	<.001
>3	59 (14.8)	45 (6.1)	23.301	<.001
Length of stent(mm)	59.31 ± 30.20	39.25 ± 25.00	−8.586^a^	<.001
Stent diameter(mm)	2.88 (2.63，3.21)	3.0 (2.75，3.25)	−3.201^a^	.001
Medicine				
Asprin(%)	377 (94.7)	700 (95.5)	0.34	.56
Clopidogrel(%)	355 (89.2)	670 (91.4)	1.482	.223
Ticagrelor(%)	25 (6.3)	40 (2.5)	0.324	.569
Beta blocker(%)	311 (78.1)	544 (74.2)	2.154	.142
ACEI/ARB(%)	262 (65.8)	470 (64.1)	0.638	.425
Statin(%)	367 (92.2)	697 (95.1)	3.833	.05
CCB(%)	150 (37.7)	296 (40.4)	0.784	.376

Abbreviations: ACEI/ARB, angiotension‐converting enzyme inhibitors/angiotensin receptor blockers; AIP, atherogenic index of plasma; BMI, body mass index; BNP, brain natriuretic peptide; CCB, calcium‐channel blockers; CRP, C‐reactive protein; CTO, chronic total occlusion; DBP, diastolic blood pressure; EF, ejection fraction; GLu, glucose; HCY, homocysteine; HDL, high‐density lipoprotein cholesterol; HGB, hemoglobin; HR, heart rate; LA, left atrium; LAD, left anterior descending; LCX, left circumflex; LDL, low‐density lipoprotein cholesterol; LM, left main; LPa, lipoprotein (a); LVEDD, left ventricular end diastolic diameter; PLT, platelet; RBC, red blood cell; RCA, right coronary artery; SBP, systolic blood pressure; TC, total cholesterol; TG, triglyceride; TP, totalprotein; UA, uric acid; WBC, white blood cell.

^a^ t values.

### 3.2 The AIP and clinical scores of the groups

The CTO group showed a significantly higher AIP than the control group (0.361 vs. 0.322 ng/ml) (all p < .05; Table 2 and Figure S1). Similarly, the GRACE, TIMI, and Gensini scores were also significantly higher in the CTO group than in the control group.

**TABLE 2:** Correlation of the AIP with clinical and biochemical parameters

	BMI	Hcy	BNP	CRP	GRACE score	TIMI score	HR	Gensini score	SBP	DBP	Stent diameter
AIP Score	0.228^a^	0.029	−0.095	0.051	0.157^a^	0.025	0.046	0.325^a^	−0.051	0.026	−0.063
	0.003	−0.034	0.128^a^	0.133^a^	0.097	0.133^a^	0.127^a^	0.194^a^	0.087	0.169^a^	0.150^a^
	LVEDD	EF	WBC	RBC	HGB	PLT	LDL	UA	Glu	TC	Length of stent

Abbreviations: AIP, atherogenic index of plasma; BMI, body mass index; BNP, brain natriuretic peptide; CRP, c‐reactive protein; DBP, diastolic blood pressure; EF, ejection fraction; GLu, glucose; HCY, homocysteine; HGB, hemoglobin; HR, heart rate; LDL, low‐density lipoprotein cholesterol; PLT, platelet; RBC, red blood cell; SBP, systolic blood pressure; TC, total cholesterol; WBC, white blood cell; UA, uric acid.

^a^ *P* < 0.05.

### 3.3 Correlations of the AIP with the scores and biochemical parameters

To further explore the associations between the AIP, scores, and biochemical parameters, we analyzed an array of correlations. The BMI (*r* = 0.228, p *<* .001), GRACE score (*r* = 0.157, p *<* .001), and Gensini score (*r* = 0.325, p *<* .001) showed significant positive correlations with the AIP (Figure S2). In addition, the AIP was significantly correlated with several biochemical parameters, such as the WBC count, red blood cell (RBC) count, and TC concentration. Furthermore, the total stent length showed a significant correlation with the AIP (*r* = 0.150, p *<* .05, Table 2).

### 3.4 ROC curve analysis of the inflammatory adipocytokines in CAD

Table 3 and Figure S3 illustrate the ROC analyses of the AIP for the diagnosis of CTO. The ROC curve showed that the AUC for the AIP was 0.763 (95% CI: 0.733–0.793), and the optimal cutoff value of the AIP was 0.345, with a Youden index of 0.438. The sensitivity of the AIP for the diagnosis of CTO was 65.6% and the specificity was 78.2%, and the positive and negative likelihood ratios were 3.01 and 0.44, respectively, with a diagnostic OR of 6.84.

**TABLE 3:** The ROC curve analysis of the AIP with CTO

Factors	AUC	p	95%CI	*Se*(%)	Sp(%)	PPV(%)	NPV(%)	LR+	LR−	OR	YI	Cutoff point
AIP	0.763	<.001	0.733–0.793	65.6	78.2	61.40%	80.00%	3.01	0.44	6.84	0.438	0.345

Abbreviations: AIP, atherogenic index of plasma; LR+, positive likelihood ratios; LR−, negative likelihood ratios; NPV, negative predictive values; OR, diagnostic odd ratio; PPV, positive predictive value; *Se*, sensitive; SP, specifity; YI, Youden index.

### 3.5 Univariate and multivariate logistic regression analyses

**FIGURE 1:**
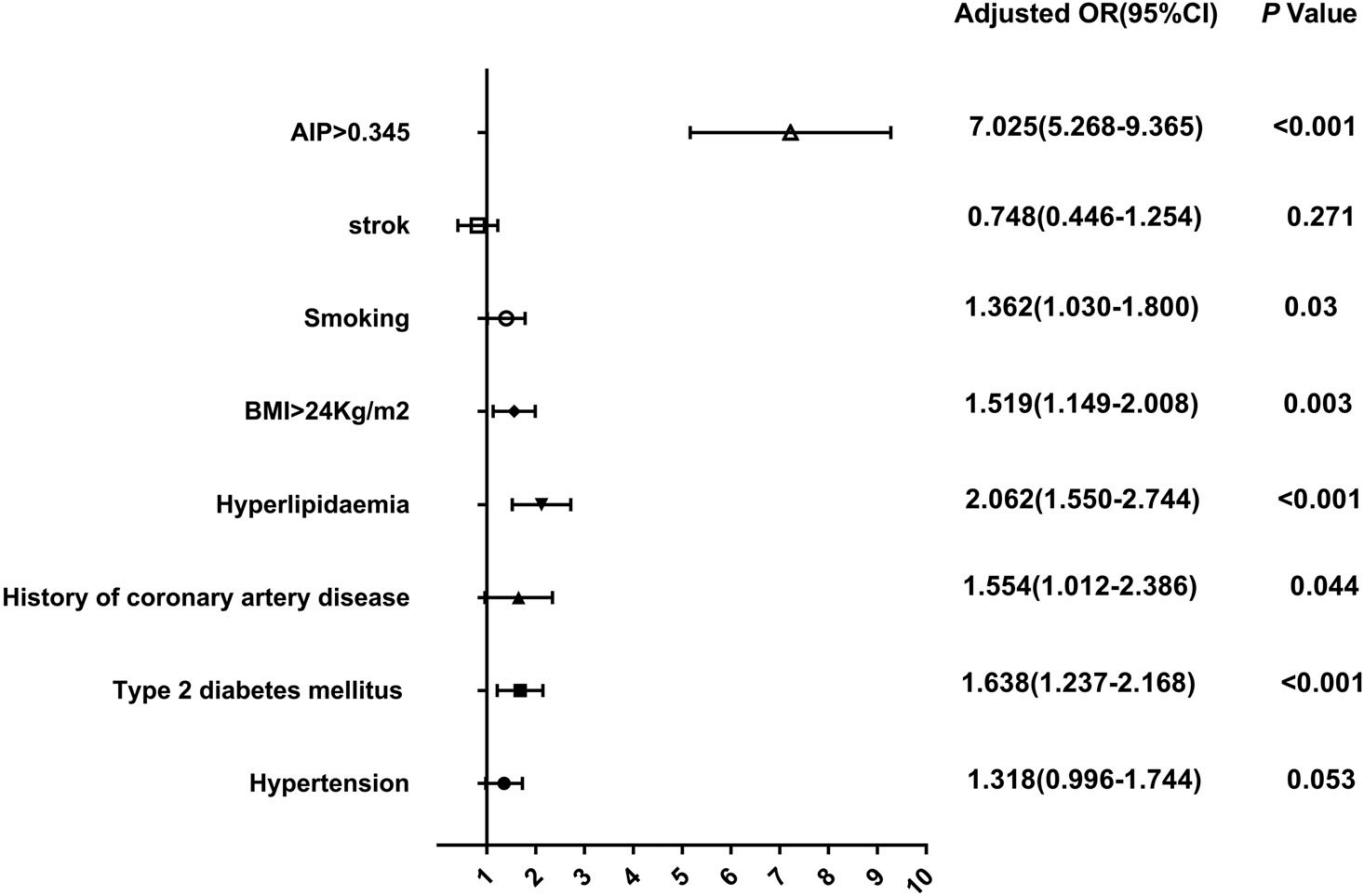
An AIP of >0.345 is independently associated with the presence of CTO. AIP, atherogenic index of plasma; CTO, chronic total occlusion

After adjustment for traditional confounders such as hypertension, type 2 diabetes mellitus, and smoking, we found that an AIP of >0.345 was independently associated with the presence of CTO (OR = 7.024, 95% CI: 5.268–9.365, Table 4, and Figure 1).

**TABLE 4:** Univariate and multivariate logistic regression analyses for AIP with CTO risk

Variables	Unadjusted odd ratio(95%CI)	p value	Multivariable adjusted odd ratio(95%CI)	p value
Hypertension	1.167 (0.914–1.491)	.216	1.318 (0.996–1.744)	.053
Type 2 diabetes mellitus	1.653 (1.290–2.117)	<.001	1.638 (1.237–2.168)	<.001
History of coronary artery disease	1.450 (0.998–2.106)	.051	1.554 (1.012–2.386)	.044
History of PCI	1.007 (0.739–1.372)	.964	—	—
EF	0.462 (0.327–0.652)	<.001	—	—
Hyperlipidaemia	1.668 (1.301–2.139)	<.001	2.062 (1.550–2.744)	<.001
BMI > 24Kg/m2	1.782 (1.392–2.280)	<.001	1.519 (1.149–2.008)	.003
Smoking	1.453 (1.136–1.857)	.003	1.362 (1.030–1.800)	.03
stroke	0.983 (0.624–1.548)	.941	0.748 (0.446–1.254)	.271
AIP > 0.345	6.405 (4.892–8.386)	<.001	7.024 (5.268–9.365)	<.001

Abbreviations: AIP, atherogenic index of plasma; EF, ejection fraction.

## 4 DISCUSSION

In this cohort study, we demonstrated significantly greater AIP values in the CTO group than in the control group based on univariable and multivariable analyses. The AIP was also significantly correlated with the Gensini and GRACE scores and showed significant associations with biochemical parameters such as the WBC and RBC counts. Thus, the AIP may provide critical clues for the diagnosis, risk evaluation, and prognostic assessment of CTO. Furthermore, the AIP, as a novel biomarker, showed diagnostic value for CTO. To the best of our knowledge, this is the first study to explore the association between the AIP and CTO.

TGs are a causal risk factor for atherosclerotic cardiovascular diseases, and many mechanisms involving both atherogenesis and thrombogenesis have been proposed. The specific markers of TG metabolism, such as lipoprotein lipase and apolipoprotein C‐III, are entrapped in the subendothelial space and scavenged by resident macrophages, contributing to macrophage foam cell formation and plaque formation and progression.^12^ Moreover, the lipolytic products of TGs also activate numerous proinflammatory, procoagulant, and proapoptotic signaling pathways that play fundamental roles in the pathogenesis of atherosclerosis.^13^ Over a 27‐year follow‐up period, TG levels >5 mmol/L (440 mg/dl) and <1 mmol/L (88 mg/dl) were associated with 17‐ and 5‐fold risks of myocardial infarction.^14^ As the smallest and densest of the plasma lipoproteins, high‐density lipoprotein (HDL) has been identified as a major recipient of free cholesterol and reversed cholesterol transport.^15^ HDL also performs functions such as the promotion of cholesterol efflux from macrophages, antioxidant activity, inhibition of vascular inflammation, and improvement of endothelial function.^16^ In the Framingham Heart Study and Prospective Cardiovascular Munster study, low HDL‐C levels were independent risk factors for cardiovascular diseases, and an increase of 1 mg/dl (0.026 mmol/L) in the HDL‐C level was associated with a 2%–3% risk reduction.^17^ As a basic pathological feature of CTO, the proximal cap is often fibrotic or calcified and is composed of fibrous tissue, atheroma, calcified tissue, and focal lymphocytic infiltration. The process of CTO is closely associated with haemorrheologic abnormalities, especially in patients with diabetes and dyslipidaemia.^18^

In this study, the AIP showed the strongest correlation with the Gensini score in all patients, demonstrating its vital role in the development of atherosclerosis. As a surrogate for small, dense low‐density lipoprotein particles, the AIP was negatively associated with the low‐density lipoprotein cholesterol particle diameter. Endothelial dysfunction and atherosclerosis are attributable to phenomena such as increased lipid peroxidation, activation of oxygen radicals, and the expression of adhesion molecules.^19^ The AIP was also proposed to be an economic and reliable indicator for these phenomena in clinical practice. Several studies have demonstrated that the AIP is associated with the development of CAD. Cai et al. proved that the AIP was significantly higher in the CAD group than in the control group, potentially making it a strong and independent predictor for CAD.^5^ In an observational study, the AIP was independently associated with the presence and severity of acute coronary syndrome in young adults.^20^ Similarly, Shen et al. suggested that the AIP was linearly correlated with waist circumference and could be used as a reference to estimate abdominal obesity, which was similar to our finding showing that the AIP was positively associated with the BMI.^21^ In another cross‐sectional survey, higher AIP values tended to be associated with a higher risk of obesity.^22^

Some recent studies have proven the substantial value of the AIP in evaluating the severity of coronary syndrome. A previous study enrolled 1437 patients without CAD and 2253 patients with CAD and revealed a positive correlation between the AIP and SYNTAX score.^23^ In another study, 1124 participants who had undergone a coronary artery calcification measurement were stratified into three groups based on the AIP values and followed up for 4.2 years. That study indicated a significant correlation between the AIP and the progression of coronary artery calcification.^24^ Furthermore, previous studies demonstrated that the AIP may be an independent predictor of CTO complexity and was associated with the numbers and lengths of stents applied after successful recanalisation in addition to the J‐CTO score.^25^ Dyslipidaemia is an important mechanism for the progression of coronary stenosis. In this study, we compared the AIP values between patients with and without CTO and discovered that the AIP was associated with disease severity and could be used to predict the presence of CTO.

The GRACE score, which has eight variables, has been validated for the prediction of major adverse cardiac events. The TIMI score derived from the TIMI IIB trial can also be used to estimate the possible severity of CAD.^26^ In our study, the AIP was significantly associated with the GRACE score, which indicated the clinical value of AIP assessments. The AIP was also a better biomarker for CAD than conventional lipid measurements since an elevated AIP was associated with cardiovascular events and all‐cause mortality. A 7.8‐year follow‐up study of 2676 middle‐aged adults confirmed the prognostic value of the AIP for predicting morbidity due to CAD.^27^ In a previous study that enrolled 500 participants, the AIP was associated with the risk of all‐cause death in the 10‐year follow‐up.^28^

Interestingly, the AIP is affected by regions, populations, and diet. In the rural areas of northeast China, the prevalence of AIP values >0.21 was 23.1%,^29^ while the AIP values were 0.46 in the Turkish population and − 0.1 among the staff of a university in Malaysia.^30^ The present findings indicate that the AIP may have diagnostic benefits in patients with CTO. Furthermore, as an economic and reliable indicator, it may be beneficial in the evaluation of the severity of coronary stenosis and the clinical outcomes. This study further confirmed the importance of the AIP in the evaluation of CAD. The AIP can facilitate the selection of interventional strategies and individualized treatment regimens and may provide new insights concerning the diagnosis and risk assessment of patients with CTO.

### 4.1 Limitations

Our study has some limitations. First, the single‐centre nature of this study and the relatively small number of enrolled patients may have introduced selection bias. Second, our data could not fully explain the physiopathologic relationship between the AIP and CTO. Furthermore, the control group included cases of intermediate and severe CAD. If we had enrolled only cases of obstructive CAD, the value of the AIP in patients with CTO would have been further confirmed. Another potential limitation is that the biomarker concentrations in our study were assessed with a single measurement.

### 4.2 Future directions

Our study findings suggest that the AIP can predict the occurrence of CTO. However, additional studies are needed to examine the mechanism of this association and determine the role of the AIP in the pathogenesis of CTO.

## 5 CONCLUSION

Our study findings suggest that the AIP is independently associated with the occurrence of CTO and could predict the presence of CTO and disease severity.

## CONFLICT OF INTEREST

The authors have no conflicts of interest to declare.

## Supporting information


**Fig. S1** Values of the AIP and clinical assessments of the two groups. (A) AIP. (B) GRACE score. (C) Gensini score. (D) TIMI score. AIP, atherogenic index of plasma; CTO, chronic total occlusion; GRACE, Global Registry of Acute Coronary Events; TIMI, thrombolysis in myocardial infarction.

**Fig. S2** Correlations among the AIP level, clinical assessments, and BMI. (A) Gensini score. (B) BMI. (C) GRACE score. (D) TIMI score. AIP, atherogenic index of plasma; BMI, body mass index; GRACE, Global Registry of Acute Coronary Events; TIMI, thrombolysis in myocardial infarction.

**Fig. S3** ROC curve analysis of the AIP for the diagnosis of CTO. AUC: 0.763, 95% CI: 0.733–0.793. AIP, atherogenic index of plasma; AUC, area under the curve; CI, confidence interval; CTO, chronic total occlusion; ROC, receiver‐operating characteristic.
