# Supplementary figures and images for: Predictive value of the atherogenic index of plasma for chronic total occlusion before coronary angiography
Source: Clin Cardiol. 2021 Mar 9;44(4):518–25. doi: 10.1002/clc.23565 (PMC8027576; doi:10.1002/clc.23565)

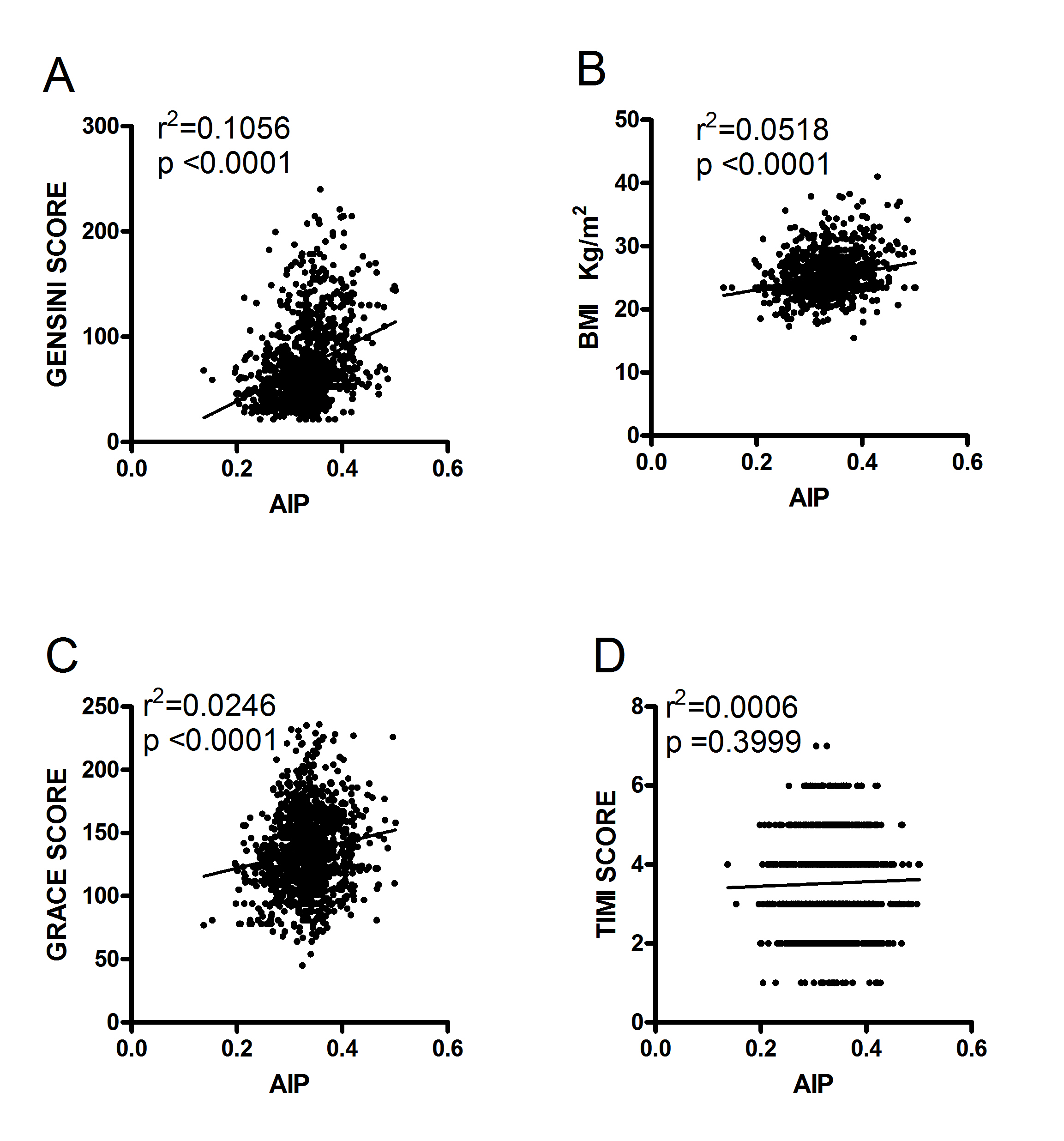

Supplement: Supplementary file 1 — Fig. S1 Values of the AIP and clinical assessments of the two groups. (A) AIP. (B) GRACE score. (C) Gensini score. (D) TIMI score. AIP, atherogenic index of plasma; CTO, chronic total occlusion; GRACE, Global Registry of Acute Coronary Events; TIMI, thrombolysis in myocardial infarction. [file CLC-44-518-s002.jpg]

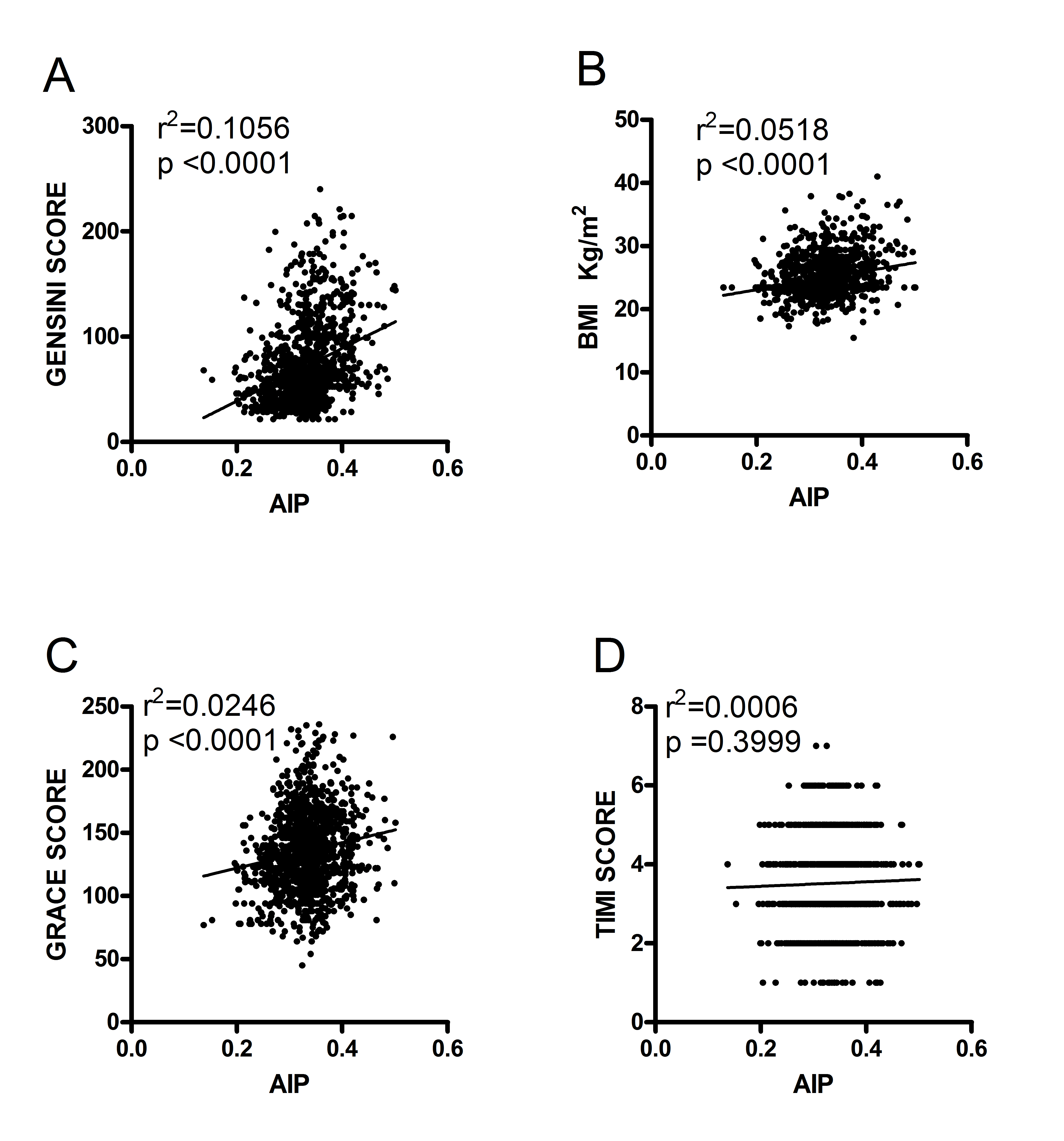

Supplement: Supplementary file 2 — Fig. S2 Correlations among the AIP level, clinical assessments, and BMI. (A) Gensini score. (B) BMI. (C) GRACE score. (D) TIMI score. AIP, atherogenic index of plasma; BMI, body mass index; GRACE, Global Registry of Acute Coronary Events; TIMI, thrombolysis in myocardial infarction. [file CLC-44-518-s003.jpg]

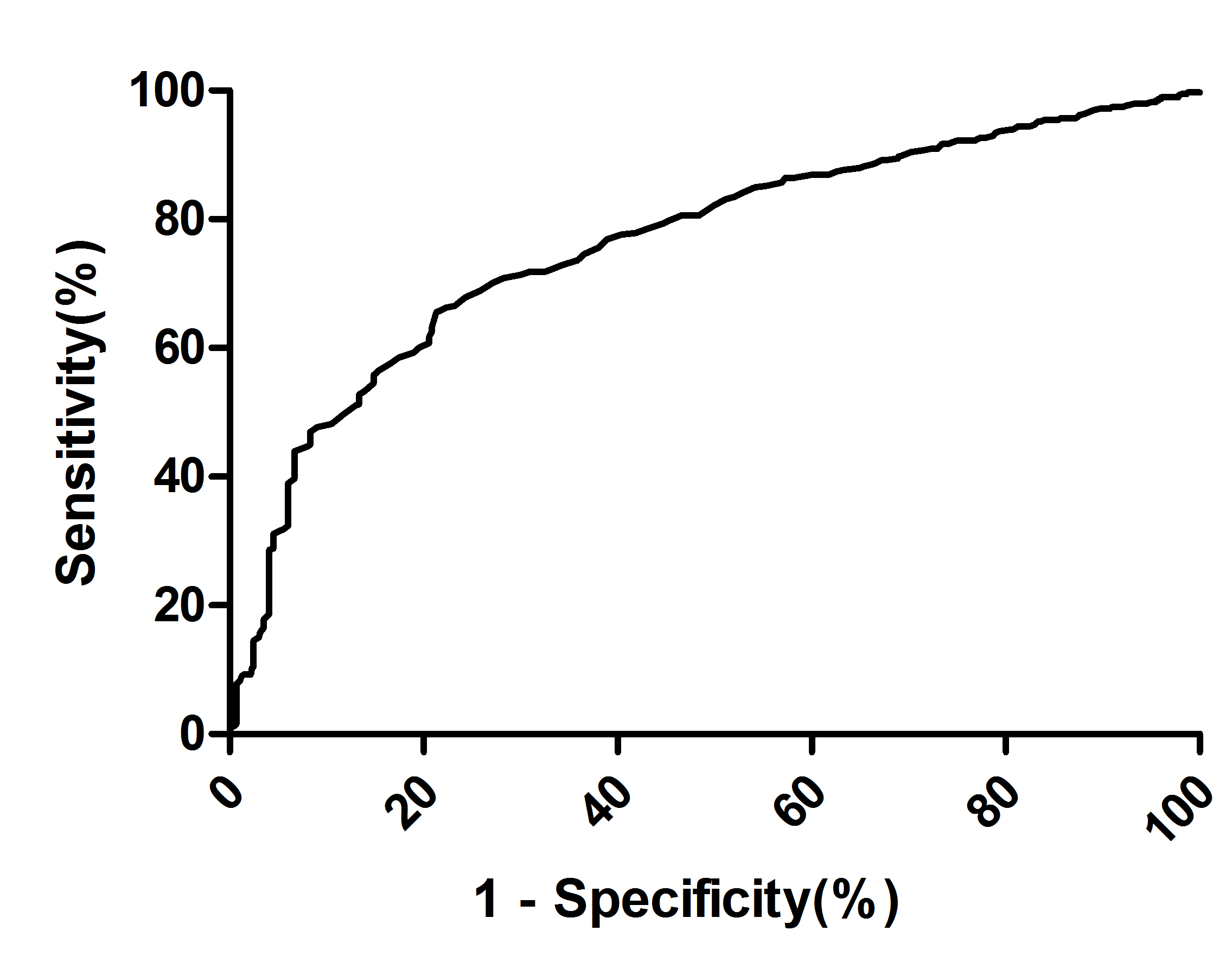

Supplement: Supplementary file 3 — Fig. S3 ROC curve analysis of the AIP for the diagnosis of CTO. AUC: 0.763, 95% CI: 0.733–0.793. AIP, atherogenic index of plasma; AUC, area under the curve; CI, confidence interval; CTO, chronic total occlusion; ROC, receiver‐operating characteristic. [file CLC-44-518-s001.jpg]
